# Preconceptional evaluation of women with recurrent pregnancy loss: the additional value of assessing vascular and metabolic status

**DOI:** 10.1186/s12884-021-04365-5

**Published:** 2022-01-27

**Authors:** Denise H. J. Habets, Veronique M. M. M. Schiffer, Lisa P. A. Kraneburg, Femke J. W. de Krom, Irem Gürtekin, Bo E. van Bree, Ron J. T. van Golde, Lotte Wieten, Marc E. A. Spaanderman, Salwan Al-Nasiry

**Affiliations:** 1 Department of Obstetrics and Gynaecology, Maastricht University Medical Centre (MUMC+), Maastricht, The Netherlands; 2 GROW School for Oncology and Developmental Biology, Maastricht University, Maastricht, The Netherlands; 3 Department of Reproductive Medicine, Maastricht University Medical Centre (MUMC+), Maastricht, the Netherlands; 4 Department of Transplantation Immunology, Maastricht University Medical Centre (MUMC+), Maastricht, The Netherlands

**Keywords:** Recurrent pregnancy loss, Cardiovascular disease, Metabolic syndrome

## Abstract

**Background:**

A majority of recurrent pregnancy loss cases (RPL) remains unexplained. We hypothesized that complications in vascular and metabolic status may guide towards underlying problems that also predispose to RPL and that the number of pregnancy losses is related.

**Methods:**

A retrospective study in 123 women with either a history of low-order RPL (2–3 pregnancy losses) or high-order RPL (≥ 4 pregnancy losses) and 20 women with a history of uncomplicated pregnancy (controls) was performed. Vascular status was assessed by measuring hemodynamic parameters, determining abnormal parameters and analyzing their contribution to the circulatory risk profile (CRP). In a similar way, metabolic status was assessed. Metabolic parameters were measured, used to determine abnormal parameters and analyzed for their contribution to the metabolic syndrome (MetS).

**Results:**

No major differences were observed in vascular or metabolic parameters between women with RPL and controls. There was no relation with the number of pregnancy losses. However, when analyzing the presence of abnormal constituents, more than 80% of women with RPL had at least one abnormal constituent of the CRP. While only 27% had one or more abnormal constituent of the MetS.

**Conclusions:**

The presence of abnormal circulatory factors prior to pregnancy, and to lesser extent constituents of the metabolic syndrome, may predispose to RPL and offer new insights to its pathophysiology.

## Introduction

Recurrent pregnancy loss (RPL) is a heterogeneous condition and a devastating complication of early pregnancy affecting approximately 1 in 50 couples who are trying to conceive. Although there is no consensus on its definition, the diagnosis of RPL is considered after two or more consecutive pregnancy losses from the time of conception until 24 weeks of gestation, according to most guidelines, such as that of the European Society for Human Reproduction and Embryology [1]. Although a few conditions have been causatively linked to the occurrence of RPL, such as antiphospholipid syndrome, uterine malformations and parental chromosomal aberrations, the exact pathophysiology is still elusive and a majority of RPL cases remains without an identifiable cause [1, 2]. In addition, several risk factors such as increasing maternal age, smoking, alcohol use, overweight and stress have been associated with the prevalence and prognosis of RPL [3]. Next to these associated conditions and risk factors, the number of previous pregnancy losses is relevant to the definition of RPL and the prediction of live birth in subsequent pregnancies. However, the contribution of the number of previous pregnancy losses to understanding the pathophysiology of RPL remains unclear [4, 5]. It is clear that the current diagnostic workup is not sufficient enough and new factors involved in this adverse obstetric complication are very desirable.

Women with obstetric complications, such as preeclampsia and gestational diabetes have been shown to have an increased risk of cardiovascular diseases (CVD) later in life [6–8]. Leading the American Heart Association to update its guidelines in 2011 to incorporate obstetric complications as risk factors for development of cardiovascular disease in women [9]. Moreover, women with RPL have an increased risk of ischemic stroke mortality [10] and a twofold higher risk of coronary heart disease [11] later in life. Hypotheses have been proposed for the association between RPL and CVD. First, a joint underlying genetic, thrombogenic, metabolic or immune defect may contribute to both RPL and CVD [12, 13]. The genetic predisposition to RPL is suggested by the finding that a positive family history of CVD is associated with a 1.6 higher risk of RPL [14]. Second, RPL itself could trigger or augment a cascade of inflammatory responses or other mechanisms causing endothelial dysfunction, that could lead to CVD if persistent [13, 15].

Next to this association with CVD, RPL is also associated with characteristics of the Metabolic Syndrome (MetS) such as high body mass index (BMI) [16]. In addition, the beneficial effect of metabolic interventions, such as metformin on early pregnancy outcomes, suggest the presence of a metabolic pathway among RPL cases [17].

Collectively, the above lines of evidence suggest that cardiovascular and metabolic complications may guide towards underlying problems that also predispose to RPL and that different vascular and metabolic phenotypes may be associated with RPL. There is limited knowledge on the prevalence and coincidence of vascular and metabolic complications in women with RPL. The present pilot study first aims to analyze vascular and metabolic status in non-pregnant women with RPL and women with a previous uncomplicated pregnancy. Secondly, this study aims to investigate the contribution of the number of pregnancy losses.

## Methods

### Study design

This retrospective study was approved by the Medical Ethical Committee of the Maastricht University Medical Centre (Maastricht UMC+) (14–4-118). Data was available for analysis from 123 women, who gave informed consent and participated in the preconceptional cardiovascular assessment program (PCVS) between 2015 and 2019. This program consists of a structured evaluation performed at least 3 months after miscarriage according to Dutch national guidelines (www.nvog.nl) of RPL and comprises thrombophilia screening, parental karyotypic evaluation, endocrine screening and ultrasound examination for uterine anomalies. Women of reproductive age, with 2 or more reported pregnancy losses before 24 weeks of gestation according to guidelines of the European Society for Human Reproduction and Embryology [1], were included. Women with additional pregnancy complications (e.g., stillbirth, intrauterine growth restriction or preeclampsia in previous pregnancy) or medical complications (e.g., autoimmune disease or kidney disease) were excluded. Women were subsequently divided into two subgroups; having 2 or 3 pregnancy losses (low-order RPL) or having ≥4 pregnancy losses (high-order RPL). Healthy women with at least one previous uncomplicated pregnancy, recruited after advertisement, served as a control group.

### Baseline characteristics

Maternal characteristics of age, weight, height and BMI (weight/height^2^) were reported. Furthermore, information on obstetric and medical history (number of pregnancies, parity, number pregnancy losses, family history of RPL, smoking habits and the use of coffee, alcohol, drugs and medication) was collected using standardized questionnaires.

### Vascular and metabolic status

#### Hemodynamic parameters

Plasma volume (PV) was measured by means of the indicator dilatation technique [18]. PV was standardized for body surface area (BSA) according to the Mosteller formula; multiplying the square root of the height (cm) by the weight (kg) divided by 3600. Mean arterial pressure (MAP), systolic and diastolic blood pressure were taken as the median out of eleven measurements that were measured every 3 min in half an hour (Carescape V100, GE Healthcare, Eindhoven, the Netherlands). Heart rate (HR), cardiac output (CO) and stroke volume (SV) were measured during cardiac ultrasonography according to the American Society of echocardiography (ECG) guidelines [19]. All images were acquired in left lateral position, after 10 min of rest to ensure stable hemodynamic variables and timed at the end of expiration. Images were recorded as ECG-gated digital loops (MAC 5500, GE Healthcare, Eindhoven, the Netherlands) and stored for offline analysis. Data was collected and analyzed offline using specific software (Xcelera, Philips, Best, the Netherlands) after completing all measurements. HR was calculated by measuring the time interval between two consecutive R peaks on the ECG. SV was calculated using the following formula: SV = π (OTD/2)2*VTI; three VTI traces were used to determine SV. CO was calculated as CO = HR*SV. Total peripheral vascular resistance (TPVR) was calculated as R = 80*MAP/CO. Uterine artery resistance was measured with a transvaginal probe (GRIC5-9D, GE Healthcare, Eindhoven, the Netherlands) by means of a previously published technique [20]. In brief, the velocity in the selected artery had to be 60 cm/s or higher to meet the standard of being the uterine artery instead of other paracervical vessels. The angle of insonation had to be as close as possible to 0° and pulsatility index (PI), calculated as peak systolic velocity - end diastolic velocity / time averaged velocity, was measured over at least three cardiac cycles in both left and right uterine artery. Mean uterine artery PI was calculated from the left and right uterine artery PI.

#### Circulatory risk profile (CRP)

The CRP was previously described by Scholten et al. [21] and was defined as 1) hypertension: systolic blood pressure of 140 mmHg or higher or diastolic blood pressure of 85 mmHg or higher or the use of antihypertensive medication; 2) reduced PV: PV less than 1405 mL/m^2^; 3) increased TPVR: TPVR more than 1600 dyne.sec/cm^5^. Women who used antihypertensive medication were not included in the calculations detailed concerning plasma volume and vascular resistance in order to prevent any confounding effect of medication; 4) increased left or right uterine artery PI: right uterine artery PI (> 2.66), or left uterine artery PI (> 2.33) were considered abnormal as these non-pregnant cut-off values were shown to be discriminatory between healthy and complicated pregnancies as previously described by Spaanderman et al. [22].

#### Metabolic parameters

Total cholesterol, high-density lipoprotein (HDL) cholesterol and triglycerides were analyzed using an enzymatic colorimetric assay (Cobas 8000 instrument, Roche Diagnostics, Mannheim, Germany). Glucose was analyzed with an enzymatic Spectrophotometric assay (Cobas 8000 instrument, Roche Diagnostics, Mannheim, Germany) and insulin with a chemiluminescent immunometric assay on the Immulite XPi instrument (Siemens Healthcare Diagnostics, New Orleans, USA).

#### Metabolic Syndrome (MetS)

The MetS was defined according to the National Cholesterol Education Program - Adult Treatment Panel III [23] as having 3 or more of the following: 1) abdominal obesity, defined as BMI > 30 kg/m^2^ [24]; 2) hypertriglyceridemia, serum level of triglycerides of ≥1.7 mmol/L; 3) low HDL cholesterol, ≤1.29 mmol/L 4) elevated blood pressure, systolic/diastolic blood pressure of ≥130/85 mmHg or use of antihypertensive medication; 5) hyperglycemia, fasting plasma glucose level of ≥6.1 mmol/L or the use of anti-diabetic medication.

### Statistics

Baseline characteristics, vascular and metabolic parameters were tested for normality by the Shapiro-Wilk test. Data are presented as median with interquartile range (continuous data) or as percentage (dichotomous data) and compared between subgroups (low- and high-order) RPL and control group by the Kruskal Wallis test (continuous data) or by the Chi-squared test (dichotomous data). To specify which group was significantly different from other groups, the Mann-Whitney-U test was used for continuous data. To test the correlation between continuous vascular and metabolic parameters and the number of pregnancy losses (trend analysis), a Pearson correlation analysis was performed. A P-for trend value below 0.05 was considered statistically significant. Abnormal parameters were additionally compared between subgroups RPL by Mann-Whitney-U tests. Overall, a *P*-value below 0.05 was considered statistically significant. All statistical analyses were conducted with IBM SPSS statistics version 25 (IBM Corp, Los Angeles, USA).

## Results

### Study population

**Fig. 1:**
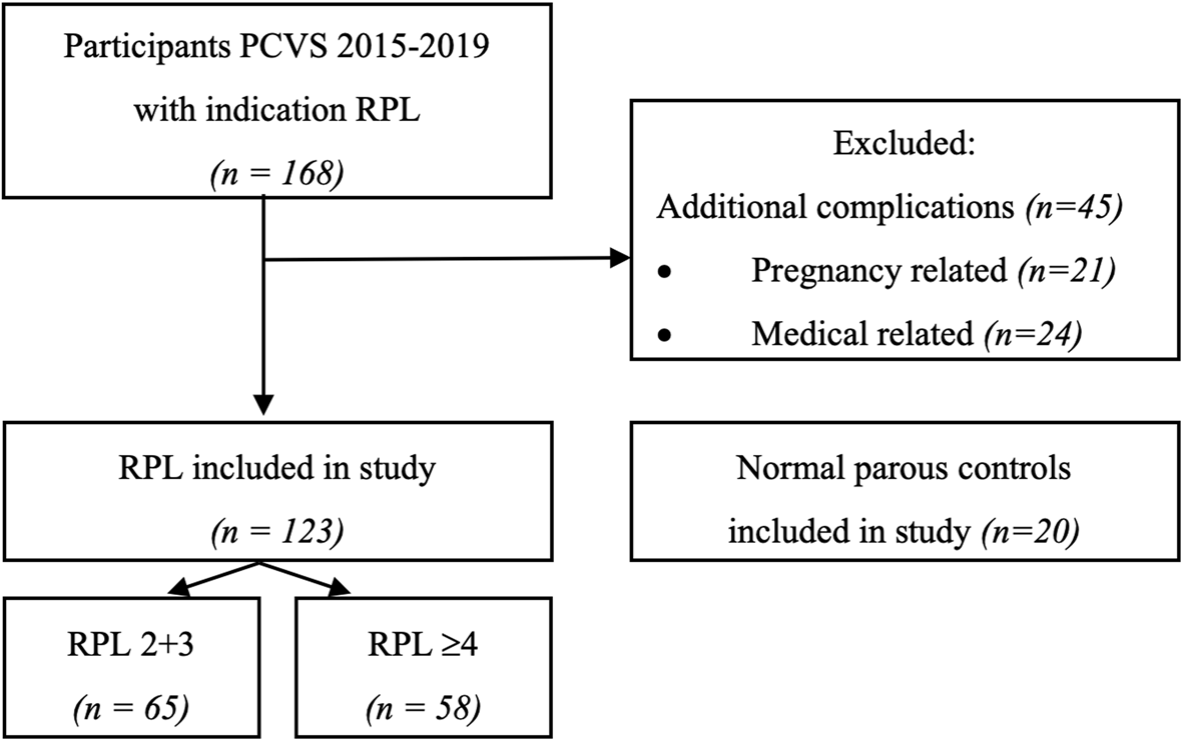
Flowchart of inclusions in study and sub-analysis PCVS, PCVS = preconceptional cardiovascular assessment program, RPL = recurrent pregnancy loss

A total of 123 women with RPL were included in the analysis; 65 with low-order RPL and 58 with high-order RPL, in addition to 20 women with previously uncomplicated pregnancies included as controls. A flowchart of inclusion is shown in Fig. 1. There were no missing values in either RPL groups, however, the control group (*n* = 20) had some missing values in baseline characteristics (Table 1). Both groups of women with RPL had more previously confirmed pregnancies vs. controls (3 [3, 4] and 5 [5–7] vs. 2 [1, 2]), more pregnancy losses (3 [2, 3] and 5 [4, 5] vs. 0 [0–1]) and fewer births (0 [0–1] and 0 [0–1] vs. 2 [1, 2]). Furthermore, both groups of women with RPL had higher body mass index vs. controls (24.4 [21.4–27.3] and 24.4 [21.7–27.3] vs. 21.8 [19.5–24.1]) kg/m^2^ (*p* = 0.028). For all other variables, no statistically significant differences were found between groups.

**Table 1:** Baseline characteristics of study population

*Baseline characteristics*	RPL = 2 + 3 (*n* = 65)	RPL ≥ 4 (*n* = 58)	Controls (*n* = 20)	*P*
Age (years)	32.8 [29.5–35.4]	33.7 [30.4–36.5]	32 [29.5–38]	0.390
BMI (kg/m^2^)	24.4 [21.4–27.3]	24.4 [21.7–27.3]	21.8 [19.5–24.1]	**0.028**
Gravida	3 [3–4]	5 [5–7]	2 [1–2]	**< 0.001**
Para	0 [0–1]	0 [0–1]	2 [1–2]	**< 0.001**
Pregnancy losses	3 [2–3]	5 [4–5]	0 [0–1]	**< 0.001**
History of				
RPL in family of patient	21.9%	35.1%	–	
RPL in family of partner	20.0%	14.0%	–	
Smoking	20.6%	24.1%	16.7%#	0.810
Alcohol use	34.9%	50.9%	41.7%#	0.210
Medication use	28.1%	25.9%	25.0%#	0.950

Data are presented as median [interquartile range] or as percentage, #9 controls had missing values - data was not available

### Vascular status

Table 2 describes the hemodynamic parameters in women with low-order RPL (2–3 pregnancy losses) versus women with high-order RPL (≥ 4 pregnancy losses) versus controls. Both groups of women with RPL differed from controls by lower mean arterial pressure (81 [76–87] and 84 [79–89] vs. 88 [81–93]), lower systolic blood pressure (107 [102–115] and 111 [105–118] vs. 116 [110–122]) and lower diastolic blood pressure (66 [59–73] and 69 [64–74] vs. 73 [68–79]). Although differences were not significant, both groups of women with RPL tended to have lower plasma volume (1433 [1313–1540] and 1405 [1307–1508] vs. 1471 [1321–1622]), higher cardiac output (4.5 [3.7–5.2] and 4.6 [4.3–5.4] vs 4.5 [4.2–4.7]), higher peripheral resistance (1425 [1275–1656] and 1474 [1231–1615] vs. 1332 [1243–1502]) and higher uterine artery PI (2.6 [2.3–3.5] and 2.7 [2.1–3.1] vs 2.5 [1.8–2.8]) compared to controls. There was no clear tendency for differences in heart rate and stroke volume between the three study groups.

**Table 2:** Comparison of non-pregnant hemodynamic parameters

*Hemodynamic parameters*	2–3 RPL (*n* = 65)	≥ 4 RPL (*n* = 58)	Controls (*n* = 20)	*P*	P for trend
Plasma volume (mL/m^2^ BSA)	1433 [1313–1540]	1405 [1307–1508]	1471 [1321–1622]	0.483	0.150
Mean arterial pressure (mmHg)	81 [76–87]	84 [79–89]	88 [81–93]	**0.006**	0.837
Systole (mmHg)	107 [102–115]	111 [105–118]	116 [110–122]	**0.003**	0.519
Diastole (mmHg)	66 [59–73]	69 [64–74]	73 [68–79]	**0.002**	0.712
Heart rate (bpm)	69 [63–77.5]	71 [65–78]	69 [62–75]	0.475	0.857
Cardiac output (L/min)	4.5 [3.7–5.2]	4.6 [4.3–5.4]	4.5 [4.2–4.7]#	0.311	0.346
Stroke volume (mL/m^2^)	67.0 [60.1–76.3]	71.8 [61.8–78.3]	71.0 [69.5–73.5]#	0.363	0.462
Peripheral resistance (dyne·s/cm^5^)	1425 [1275–1656]	1474 [1231–1615]	1332 [1243–1502]#	0.634	0.655
Mean uterine artery PI	2.6 [2.3–3.5]	2.7 [2.1–3.1]	2.5 [1.8–2.8]	0.204	0.490

Comparison of non-pregnant hemodynamic parameters between low-order RPL (2–3 pregnancy losses) high-order RPL (≥ 4 pregnancy losses) and controls, PI = pulsatility index, data are presented as median [interquartile range], #15 controls had missing values

Trend analysis for the number of pregnancy losses in RPL showed no statistical significance in hemodynamic parameters. However, with an increasing number of pregnancy losses, a lower PV (P for trend = 0.150) and CO (P for trend = 0.346) and higher SV (P for trend = 0.462) and mean uterine artery PI (P for trend = 0.490) were observed.

**Fig. 2:**
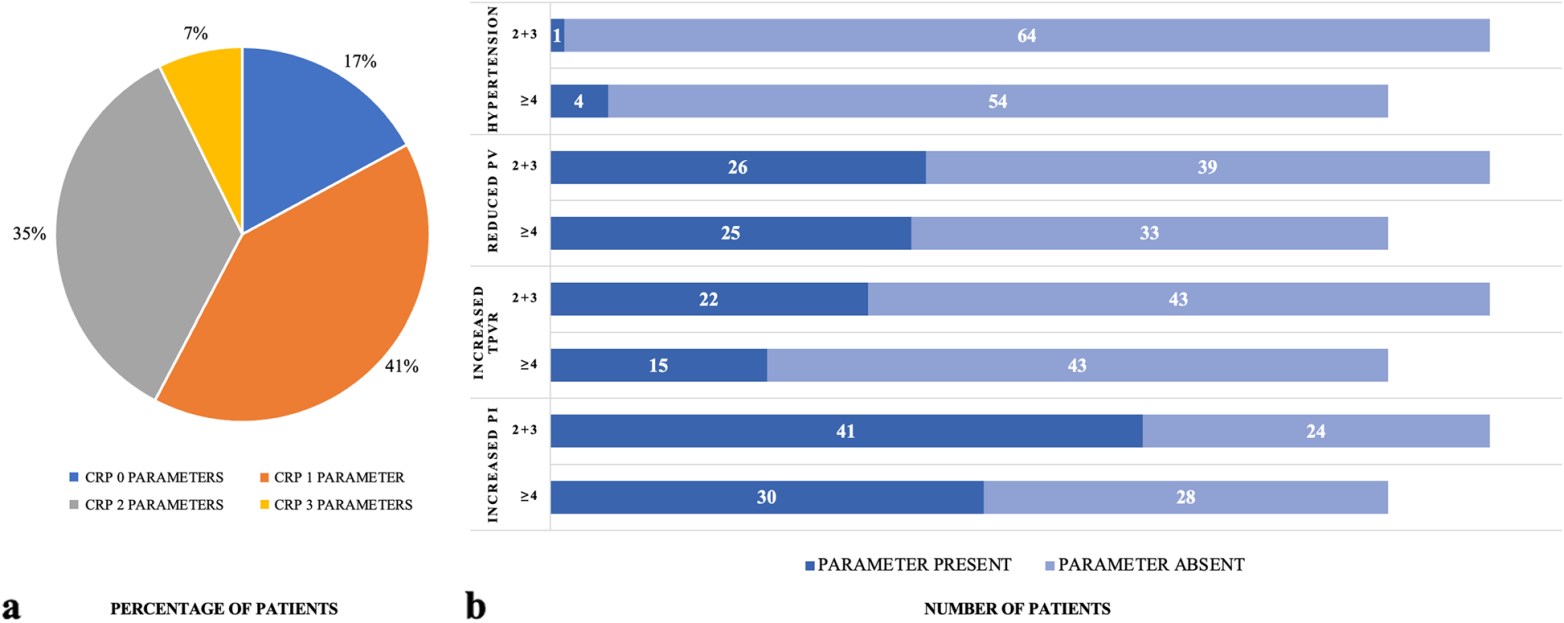
Prevalence of the circulatory risk profile in women with RPL (*n* = 123) (**a**) and its individual abnormal parameters in low-order (*n* = 65) and high-order (*n* = 58) RPL (b) CRP = circulatory risk profile PV = plasma volume TPVR = total peripheral vascular resistance PI = pulsatility index data is presented as percentage of patients (**a**) or as absolute number of patients (**b**) data of controls is not shown

Figure 2 describes the prevalence of the CRP in the total group of women with RPL (*n* = 123) and the prevalence of individual parameters of abnormal vascular status in women with low- vs. high-order RPL. Within women with RPL, 83% had at least one abnormal parameter contributing to the CRP and 42% had at least 2 abnormal parameters (Fig. 2a). There were no significant differences between the two RPL groups in individual abnormal parameters (data from controls are not shown because of missing values). Overall, hypertension was not frequently observed in women with RPL (1/65 and 4/58). Increased mean uterine artery PI was, however, commonly present (41/65 and 30/58) in women with RPL (Fig. 2b).

### Metabolic status

Table 3 describes the metabolic parameters in women with low-order RPL versus women with high-order RPL versus controls. Although there were no statistically significant differences between the three groups, women with RPL tended to have higher HDL cholesterol (1.6 [1.4–1.8] and 1.6 [1.3–1.9] vs 1.4 [1.2–1.7]), higher triglycerides (0.8 [0.7–1.1] and 0.9 [0.6–1.1] vs 0.7 [0.6–0.8]) and lower insulin (39.7 [26.9–57.6] and 36.4 [24.3–65.7] vs 46.5 [29.5–49.3]) levels compared to controls. For total cholesterol and glucose levels there was no clear tendency.

**Table 3:** Comparison of non-pregnant metabolic parameters

*Metabolic parameters*	2–3 RPL (*n* = 65)	≥ 4 RPL (*n* = 58)	Controls (*n* = 20)	*P*	P for trend
Cholesterol total (mmol/L)	4.5 [4.0–5.1]	4.5 [4.0–5.1]	4.5 [4.3–5.1]#	0.919	0.328
Cholesterol HDL (mmol/L)	1.6 [1.4–1.8]	1.6 [1.3–1.9]	1.4 [1.2–1.7]#	0.317	0.466
Triglycerides (mmol/L)	0.8 [0.7–1.1]	0.9 [0.6–1.1]	0.7 [0.6–0.8]#	0.252	0.940
Glucose fasting (mmol/L)	5.0 [4.75–5.4]	4.95 [4.8–5.3]	4.9 [4.75–5.15]#	0.730	0.657
Insulin (pmol/L)	39.7 [26.9–57.6]	36.4 [24.3–65.7]	46.5 [29.5–49.3]#	0.939	0.418

Comparison of non-pregnant metabolic parameters between low-order RPL (2–3 pregnancy losses) high-order RPL (≥ 4 pregnancy losses) and controls, HDL = high density lipoprotein, data are presented as median [interquartile range], #12 controls had missing values

Trend analysis for the number of pregnancy losses in RPL showed no statistically significance in metabolic parameters. However, total cholesterol (P for trend = 0.328) and HDL cholesterol (P for trend = 0.252) tended to be higher and insulin (P for trend = 0.418) tended to be lower as the number of losses increased.

**Fig. 3:**
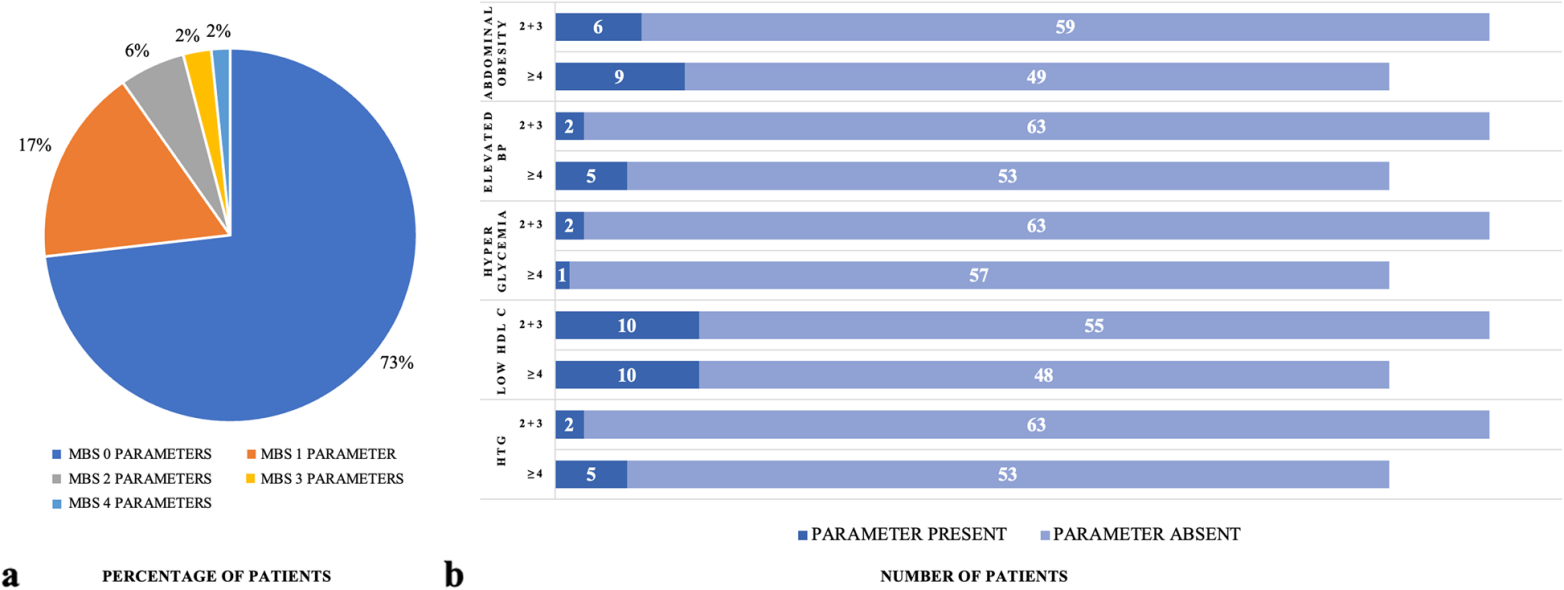
Prevalence of the metabolic syndrome in women with RPL (*n* = 123) (**a**) and its individual abnormal parameters in low-order (*n* = 65) and high-order (*n* = 58) RPL (b) MBS = metabolic syndrome BP = blood pressure HDL C = high density lipoprotein cholesterol HTG = hypertriglyceridemia data is presented as percentage of patients (**a**) or as absolute number of patients (**b**) data of controls is not shown

Figure 3 describes the prevalence of the MetS in the total group of women with RPL (*n* = 123) and the prevalence of individual parameters of abnormal metabolic status in women with low- vs. high-order RPL. Within women with RPL, 73% had none of the parameters of the MetS and 4% had 2 or 3 parameters. Therefore, 5 women with RPL were diagnosed with MetS (Fig. 3a). There were no significant differences between the two RPL groups in individual abnormal metabolic parameters (data from controls are not shown because of missing values). Overall, hyperglycemia was not frequently observed in women with RPL (2/65 and 1/58). Low HDL cholesterol was, however, more present (10/65 and 10/58) in women with RPL (Fig. 3b).

## Discussion

### Main findings

In this moderate-size retrospective analysis of 123 women with RPL undergoing extensive preconceptional assessment program there were no major differences in vascular or metabolic parameters compared to controls with previously uncomplicated pregnancies, nor among women with low- vs. high-order RPL. The number of previous pregnancy losses did not relate to vascular nor metabolic parameters. Importantly, the majority of women with RPL (83%) had at least one abnormal vascular parameter contributing to the CRP and 42% had at least 2 abnormal vascular parameters, while only 27% had an abnormal metabolic parameter of the MetS. Although high resistance in the uterine arteries was observed in the majority of women with RPL (71/123), overt hypertension was not frequently reported (5/123). These findings do not confirm our hypothesis of a dose-dependent biological relation between the number of miscarriages and vascular or metabolic status in women with RPL. The high prevalence of the CRP in these women does warrant further investigation into the role of cardiovascular system in RPL.

### Strengths and limitations

The strength of our study is the extensive assessment program of various parameters pertaining to the vascular and metabolic system and subsequently using previously described cut-off values to define abnormal parameters in these systems. In this way risk profiles were composed to facilitate interpretation of screening results and documentation of the presence of abnormal circulatory and metabolic profiles. Additionally, we took into consideration the important co-variable: the number of previous pregnancy losses, to link the severity of this adverse pregnancy complication to the vascular and metabolic system. Furthermore, in order to study a homogenous population of RPL, we excluded women with additional medical and obstetric complications as this could be an important confounder on the studied parameters. However, as hemodynamic and metabolic factors could also influence explained RPL, generalizability is limited. Finally, our analysis of the circulatory profile included data on uterine artery PI as an indication of uterine perfusion, hereby assessing not only systemic but also local vascular status, which is arguably more relevant to the pathogenesis of RPL.

There were a few limitations of this study. First, the control group was modest in size and data were not available for analysis on some parameters, decreasing the power of the study and increasing possible selection bias. However, results did not differ after stochastic regression imputation. Secondly, the indication of repeated pregnancy loss was self-reported and lacked important detailed information (e.g., ultrasound features or gestational age) to characterize well-defined subgroups of women with RPL.

### Interpretation

Preconceptional assessment of maternal vascular status might be informative on the projected success of subsequent pregnancy and is therefore a potential supplement to current investigations for women with unexplained RPL. Early pregnancy is characterized by a wide range of substantial hemodynamic and vascular changes, such as increases in maternal cardiac output associated with an increase in stroke volume and heart rate, increases in plasma volume, decreases in blood pressure and a reduction in peripheral resistance [25]. Importantly, women with reduced plasma reserves are less capable of mobilizing volumes of blood in response to increased arterial demands, as is the case in pregnancy, which in turn can be insufficient for the growing demands of the developing placenta and fetus. Our data is not consistent, though tends to be in line with previous results from a study by Donckers et al. [26], showing low plasma volumes in women with unexplained RPL. The observed trend of lower plasma volume with increasing number of previous RPL observed in our study, although not significant, supports the hypothesis that women with RPL may need sustained increased sympathetic tone to meet the arterial demands of early pregnancy. As failing to do so could be a detrimental factor contributing to pregnancy loss [26]. Alternatively, the lack of statistical significance in our study could be attributed to differences in plasma volume measurements or the choice of control group. Higher values of plasma volume were described in the nulliparous control group in the study by Donckers et al., compared to our multiparous control group. Future studies utilizing non-invasive, reproducible and readily methods for measuring plasma volume are greatly anticipated.

In addition to measurements of plasma volume, complete assessment of maternal hemodynamic status takes into account the complex interaction between the cardiac, vascular and local uterine compartments. We observed high prevalence of the CRP (83%), with a predominance of increased uterine artery PI in the majority of women with RPL, in the absence of overt hypertension. This suggests that subtle changes in uterine perfusion could be linked to inadequate adaptation of the local uterine environment in early pregnancy and could be an additional pathway to explore in the research on unexplained RPL. Although we failed to demonstrate a dose-dependent gradient between the number of miscarriages and circulatory abnormalities in women with RPL, larger studies taking into account the genetic constitution of the embryo and the gestational age at miscarriage, are better suited to elucidate the contribution of the number of previous losses to various phenotypes of RPL.

Next to vascular status, assessment of metabolic status can yield valuable information on the background of maternal constitutional factors and risks in subsequent pregnancy. Early pregnancy can be viewed as an anabolic state with an increase in maternal fat stores and small increases in insulin sensitivity, accordingly nutrients are stored to meet the demands of late pregnancy [27]. In contrast, late pregnancy is better characterized as a catabolic state with decreased insulin sensitivity and increased insulin resistance, which in turn results in increases in maternal glucose and free fatty acid concentrations, allowing greater substrate availability for fetal growth [27]. Our data implies that women with RPL do not differ significantly in preconceptional levels of cholesterol, triglycerides, glucose and insulin from women whom had a previous successful pregnancy. Furthermore, only 5 out of 123 women in our study fulfilled the criteria for the diagnosis of the MetS. This is not in line with a recent study from Hilali et al. [16] were the percentage of the MetS or of at least having one of its components was 24.4% in patients with unexplained RPL and this was associated with increasing numbers of previous pregnancy losses. The difference in our findings could be attributed to differences in average BMI: this was 26 in the patient population of Hilali et al. (considered overweight) versus 24 (considered normal weight) in our study population.

BMI was significant higher in our women with RPL and this might have had a confounding effect on hemodynamic and metabolic parameters, as plasma volume, cardiac output and peripheral resistance have proven to be positively correlated with BMI. However, as the BMI of our women was within clinical ranges of normal weight, standard deviations were similar to controls and when plasma volume, cardiac output and peripheral resistance were adjusted for body composition results remained similar, confounding contribution was considered to be minimal.

The relevance of our findings to clinical practice can be highlighted by observations from the several studies showing that vascular and metabolic abnormalities in pregnant and non-pregnant women can be treated with help of lifestyle interventions, which are relatively easy to implement in practice [28, 29]. Improved lifestyle will result in more favorable parameters and therefore increase the chance for a healthy pregnancy [29]. The risk of the MetS and CVD later in life could decrease simultaneously and therefore might have the dual benefit of improving women’s general health and fulfilling the aspiration of a healthy ongoing pregnancy.

## Conclusion

In this retrospective study we assessed vascular and metabolic status of women with a history of RPL. Although no major differences were observed in vascular or metabolic parameters between women with RPL and controls and there was no relation with the number of pregnancy losses, more than 80% of women with RPL had at least one abnormal parameter of the CRP, while only 27% had an abnormal parameter of the MetS. We discuss the findings in the context of abnormal adaptation as a potential mechanism in RPL and emphasize the relevance of preconceptional vascular and metabolic assessment and prospective trials in women with RPL.

## Data Availability

The datasets generated during and/or analysed during the current study are available from the corresponding author on reasonable request.

## Abbreviations

MUMC+: Maastricht University Medical Centre +
RPL: Recurrent pregnancy loss
CRP: Circulatory risk profile
MetS: Metabolic syndrome
CVD: Cardiovascular diseases
BMI: Body mass index
PCVS: Preconceptional cardiovascular assessment program
PV: Plasma volume
BSA: Body surface area
MAP: Mean arterial pressure
HR: Heart rate
CO: Cardiac output
SV: Stroke volume
ECG: Echocardiography
TPVR: Total peripheral vascular resistance
PI: Pulsatility index
HDL: High-density lipoprotein
